# Large-Scale Structural Analysis of the Classical Human Protein Tyrosine Phosphatome

**DOI:** 10.1016/j.cell.2008.11.038

**Published:** 2009-01-23

**Authors:** Alastair J. Barr, Emilie Ugochukwu, Wen Hwa Lee, Oliver N.F. King, Panagis Filippakopoulos, Ivan Alfano, Pavel Savitsky, Nicola A. Burgess-Brown, Susanne Müller, Stefan Knapp

**Affiliations:** 1 University of Oxford, Structural Genomics Consortium, Old Road Campus Research Building, Roosevelt Drive, Headington, Oxford, OX3 7DQ, UK; 2 University of Oxford, Department of Clinical Pharmacology, Old Road Campus, Roosevelt Drive, Oxford OX3 7DQ, UK

**Keywords:** SIGNALING, PROTEINS

## Abstract

Protein tyrosine phosphatases (PTPs) play a critical role in regulating cellular functions by selectively dephosphorylating their substrates. Here we present 22 human PTP crystal structures that, together with prior structural knowledge, enable a comprehensive analysis of the classical PTP family. Despite their largely conserved fold, surface properties of PTPs are strikingly diverse. A potential secondary substrate-binding pocket is frequently found in phosphatases, and this has implications for both substrate recognition and development of selective inhibitors. Structural comparison identified four diverse catalytic loop (WPD) conformations and suggested a mechanism for loop closure. Enzymatic assays revealed vast differences in PTP catalytic activity and identified PTPD1, PTPD2, and HDPTP as catalytically inert protein phosphatases. We propose a “head-to-toe” dimerization model for RPTPγ/ζ that is distinct from the “inhibitory wedge” model and that provides a molecular basis for inhibitory regulation. This phosphatome resource gives an expanded insight into intrafamily PTP diversity, catalytic activity, substrate recognition, and autoregulatory self-association.

## Introduction

Protein tyrosine phosphorylation is a dynamic process governed by the balanced action of tyrosine kinases and protein tyrosine phosphatases (PTPs) and is a critical event in the regulation of numerous physiological processes (Pao et al., 2007; Tonks, 2006) . Dysregulation of PTPs is associated with a multitude of diseases, and many members of the PTP family have been recognized as potential therapeutic targets (Tautz et al., 2006).

The human genome contains 107 PTPs, with the class I cysteine-based PTPs constituting the largest group. This group can be further subdivided into 61 dual-specificity phosphatases and 38 tyrosine-specific PTP genes, the “classical PTPome,” which are the focus of this study. Classical PTPs have been further subdivided into receptor (R1–R8) and nontransmembrane (NT1–NT9) subgroups (Alonso et al., 2004; Andersen et al., 2001). Twelve receptor PTPs have two catalytic domains (tandem domains), while the remaining PTPs all have a single catalytic phosphatase domain. In tandem-domain receptor protein tyrosine phosphatases (RPTPs), it is the PTP (D1) domain adjacent to the plasma membrane that displays catalytic activity while the PTP (D2) domain is either inactive or has negligible catalytic activity (Andersen et al., 2001). The functional role of the D2 domain has not yet been defined although possible roles in regulating RPTP stability, specificity, and dimerization have been suggested. Furthermore, all RPTPs, with the exception of RPTPα and RPTPɛ, contain large and diverse extracellular regions that regulate cell contacts and adhesion (Aricescu et al., 2007).

The ∼280 residue PTP catalytic domain consists of an α/β structure, and biochemical and structural studies have led to a detailed understanding of the catalytic mechanism (Barford et al., 1994; Zhang, 2002). Key features of the domain include the PTP signature motif, the mobile Trp-Pro-Asp “WPD” loop that in the closed conformation positions the conserved and catalytically important aspartate residue, and the phosphotyrosine recognition loop.

PTPs exhibit exceptional substrate specificity in vivo, which is conveyed both by the catalytic domain and other regulatory mechanisms including restricted subcellular localization, posttranslational modification events (e.g., phosphorylation), specific tissue distribution, and accessory or regulatory domains (e.g., KIM motifs or SH2 domains) (Tiganis and Bennett, 2007). Within the catalytic domain, the phosphotyrosine recognition loop (between α1–β1) contributes to the selective recognition of phosphotyrosine over phosphoserine/threonine, and two nonconserved residues following the conserved tyrosine in this loop are important in substrate interactions. A secondary phosphotyrosine (pY) binding site, proximal to the active site, has been identified in PTP1B. The secondary site is accessed from the active site via a channel referred to as the “gateway” region (Peters et al., 2000; Puius et al., 1997; Salmeen et al., 2000). The secondary pY-binding pocket has enabled the development of high-affinity inhibitors that target both pY-binding sites (Tautz et al., 2006).

A variety of mechanisms for regulation of PTP function have been reported, including inactivation of receptor PTPs by dimerization, reversible oxidation, and regulation via extracellular ligands and phosphorylation (Tonks, 2006). Dimerization as a control mechanism was first suggested based on structural studies on the membrane-proximal D1 domain of RPTPα and EGF receptor/CD45 chimeras (Bilwes et al., 1996; Desai et al., 1993). The structure revealed a dimeric assembly in which an inhibitory N-terminal helix-turn-helix wedge motif occluded the active site of the interacting catalytic domain. Subsequently, RPTPα and a number of other PTP domains have been shown to homodimerize at the cell surface resulting in inactivation (Jiang et al., 2000).

Here we report the crystal structures of 22 PTP domains, including two tandem-domain RPTP structures and a detailed structural comparison of this protein family. The generated structures include at least one member of each PTP subgroup and thereby provide a comprehensive coverage of the classical phosphotyrosine-specific PTPome. This phosphatome resource gives an expanded insight into intrafamily PTP diversity, catalytic activity, substrate recognition, and autoregulatory self-association and supports a “head-to-toe” dimerization model for RPTPγ/ζ that provides a molecular basis for inhibitory regulation of this subgroup.

## Results

**Figure 1:**
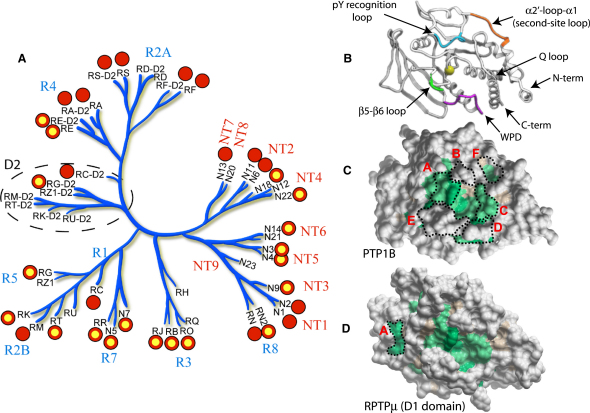
Structural Coverage of the PTPome and Surface Diversity (A) Phylogenetic tree of human PTP D1 and D2 domains indicating crystal structures determined (SGC Oxford structures are highlighted in yellow). Details of other PTP structures can be found at http://www.sgc.ox.ac.uk/research/phosphatases. PTPs are grouped into receptor PTPs (groups R1–R8) and nontransmembrane PTPs (groups NT1–NT9). The abbreviated Human Genome Organisation (HUGO: http://www.genenames.org/) gene symbol nomenclature is used in the tree, and the corresponding common names and PDB codes are provided in Supplemental Data. (B) Ribbon diagram of PTP1B with labeling of key secondary structural elements. The Cα of the catalytic cysteine residue is shown as a space-filling CPK model. (C) Conserved residues from a structure-based alignment of nonreceptor PTPs mapped onto the surface of PTP1B. Green: highly conserved, light brown: conserved residue properties only, and gray: nonconserved. (D) Conserved residues from a structure-based alignment of receptor PTPs mapped onto the surface of RPTPμ.

In this study we used a large-scale structural comparison to identify shared as well as target-specific structural features of members of the classical PTP family. A prerequisite for a conclusive structural intrafamily comparison is a comprehensive coverage of the analyzed protein family. To this end, we determined 22 catalytic domain structures of the human PTP family including 16 not published previously (Supplemental Data available online). Combined with other structure determination efforts (see http://ptp.cshl.edu/ and Almo et al., 2007), the available structures now provide high-resolution information for at least one member of each of the main receptor and nontransmembrane subgroups across the classical PTPome. The catalytic domains of the remaining PTPs of unknown structure are more than 49% identical in sequence to their closest structure-determined family member allowing the construction of reliable homology models. The structural coverage of the PTP family is outlined in the phylogenetic tree shown in Figure 1A. We also report here structures of two tandem-domain RPTPs, RPTPɛ and RPTPγ, revealing the domain organization for the PTP subgroups R4 and R5. The novel structures discussed here were refined at high resolution (average resolution of 2.0 Å and satisfactory stereochemistry; Supplemental Data).

Examination of the constructed phylogenetic tree (Figure 1A) revealed that the D2 domains from the R2A and R4 subgroups are most closely related to their respective D1 domains, suggesting a gene duplication event, while the D2 domains from RPTPγ, RPTPζ, RPTPμ, RPTPκ, RPTPρ, and RPTPλ cluster independently of their D1 domains and appear to have a distinct evolutionary root.

Superimposition of all known individual PTP D1 and D2 domain structures showed that PTPs fold into a single domain of β sheets flanked by α helices and have a highly conserved topology. The key structural features are highlighted in Figure 1B. Superimposition of all five tandem-domain RPTP structures (CD45, LAR, RPTPσ, RPTPγ, and RPTPɛ) showed that the orientation of the D1 and D2 domains is highly conserved and root mean square deviation (rmsd) values of less than 4.4 Å were obtained, for each pairwise comparison, considering Cα positions of both domains (Supplemental Data).

### PTPs Have Very Diverse Surface Properties

In contrast to the conserved ternary structure, the surfaces of PTPs are surprisingly diverse. A structure-based alignment of all experimental structures for nontransmembrane and receptor PTPs was used to map conserved residues onto the surface of PTP1B and the D1 domain of RPTPμ, respectively (Figures 1C and 1D). This mapping of surface residues identified only a few conserved surface patches comprising loop regions surrounding the active site (Figure 1C, labels A and C). Conserved surfaces are formed by the phosphotyrosine recognition loop (KNRY motif) and by conserved glutamine residues of the Q loop. Conserved WPD loop residues are largely buried (Figure 1C, label D). Highly diverse regions including the two residues following the KNRY motif (Figure 1C, label B) and the topology of the secondary substrate-binding pocket present in a number of PTPs (Figure 1C, label F) contribute to defining substrate specificity. A conserved tyrosine residue (Tyr1019 in RPTPμ) flanking the active site is characteristic for RPTPs (Figure 1D, label A), and the face opposing the active site contains only one conserved cleft as already noted (Andersen et al., 2001).

**Figure 2:**
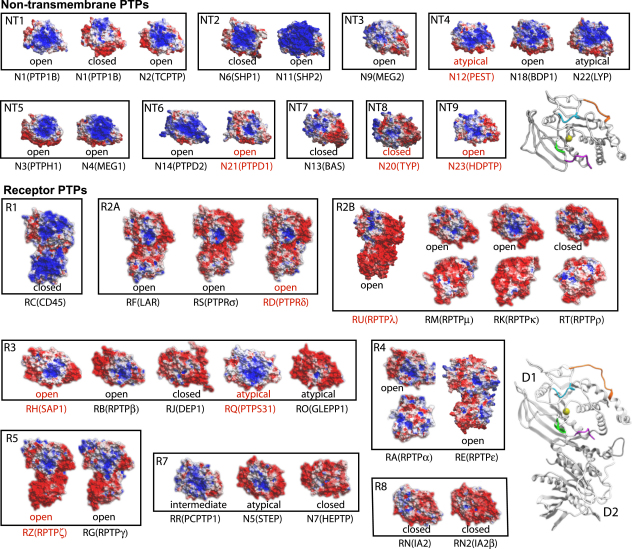
Diversity in Surface Electrostatic Potential across the PTPome Surface representations showing the calculated electrostatic potential (rendered in ICM) of PTP family members from crystal structures (black) and homology models (red). The colors of surface elements were capped at ±3 kcal/electron units (+3 = blue; −3 = red) when the calculated potentials were transferred to the surface. The WPD loop conformation is indicated under each structure.

The diverse PTP surface gives rise to significant differences in surface electrostatic potential, a property that is likely to influence substrate recognition, association with regulatory proteins, and regulatory mechanisms such as dimerization (Figure 2). This diversity, which was first noted by Alonso et al. based on homology models (Alonso et al., 2004), is apparent not only between different PTP family members but also within individual subgroups. However, some diversity in surface potential is introduced by differences in the open or closed states of the active site (e.g., compare PTP1B open/closed conformations; Figure 2), but such changes are localized only to a small surface area. Particularly notable within the RPTP group is the significantly different electrostatic potentials of CD45 and either LAR or RPTPγ. The active site and proximal surface in most PTPNs are largely electropositive, whereas in most RPTPs areas of positive electrostatic potential are mainly localized to the active sites.

### Catalytic Loop (WPD) Movement

**Figure 3:**
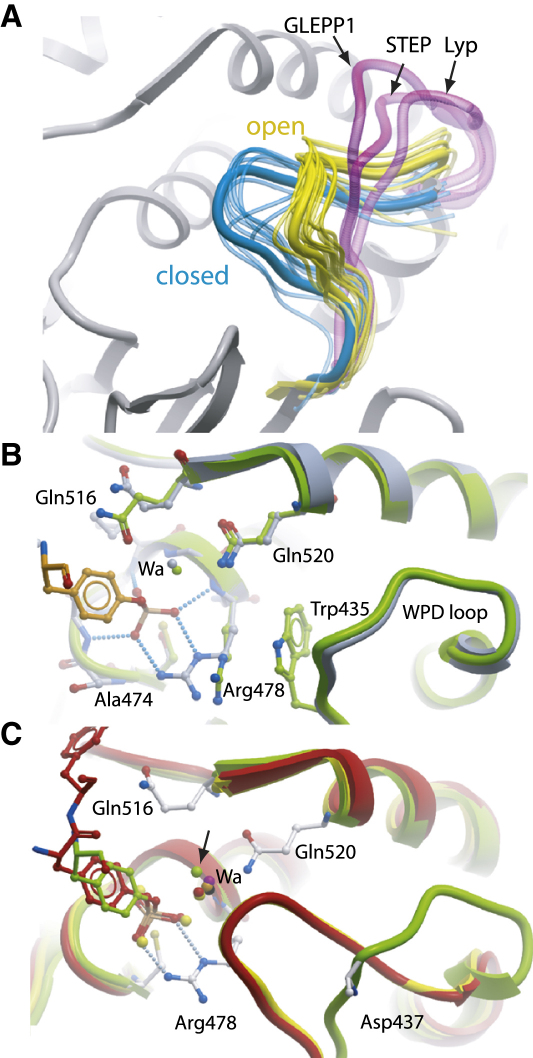
Novel Conformations and Movement of the Catalytic (WPD) Loop (A) WPD loop conformations are shown by a PTP representative of each state: closed (blue, PTP1B, PDB: 1SUG); open (yellow, PTP1B, PDB: 2HNP); and atypical (magenta, GLEPP1, PDB: 2GJT; STEP, PDB: 2BIJ; Lyp, PDB: 2P6X). The intermediate WPD loop conformation of PCPTP1 (PDB: 2A8B) is not shown for clarity. Other PTP structures are shown with a thin transparent line tracing the backbone and are colored according to conformation. (B) Superimposition of the structure of STEP-C/S in complex with pY (PDB: 2CJZ; gray) and the apo STEP (PDB: 2BIJ; light green) showing that the WPD loop conformation does not change on substrate binding (pTyr, orange). The catalytic water molecule (Wa) corresponding to that found in closed structures is shown. (C) Superimposition of the structure of STEP-C/S in complex with pY (PDB: 2CJZ; green) and PTP1B with the insulin receptor peptide (PDB: 1G1H; red). The conserved water molecule found in closed structures is shown: PTP1B (1SUG, yellow); GLEPP1 (2G59, orange); HePTP (2A3K, black), DEP1 (2NZ6, magenta). The arrow indicates the position of the displaced water molecule in STEP-C/S structure.

The dynamics of the opening/closing transition of the WPD loop has implications for PTP substrate recognition and catalytic efficiency and is also of paramount importance for the development of selective PTP inhibitors that may recognize a certain loop conformation. Structural comparisons of all available PTP structures identified four main WPD loop conformations: a closed state, an intermediate state, an open state, and an atypically open state present in STEP, LYP, and GLEPP1 (Figure 3A). The atypically open state is associated in STEP with a stabilizing 3_10_ helix C-terminal to the WPD loop (Eswaran et al., 2006) and in LYP and GLEPP1 an extra turn of helix α3 following the WPD loop. The presence of an atypically open state, catalytically nonactive conformation, in three PTPs from different subgroups suggests that this conformation may have significance for the family as a whole.

On cocrystallization of a substrate-trapping mutant of STEP (Cys472Ser) with pY bound to the active site, the WPD loop was also present in the atypical open state (Figure 3B), indicating that this conformation is stable and that substrate binding alone is not sufficient to induce loop closure as initially suggested based on apo structures and substrate complexes of PTP1B (Barford et al., 1998). The observation of an open substrate complex and closed apo structures (Pedersen et al., 2004) raises the question of the mechanism that triggers loop closure. Structural comparison revealed that all closed structures share a tightly bound “catalytic water” molecule coordinated by two conserved glutamine residues (Gln262 and 266 in PTP1B) (Figure 3C). In PTP1B this conserved water molecule has been noted previously in a closed apo-structure, in addition to three water molecules that mimic the presence of oxygen atoms of a substrate phosphotyrosine (Pedersen et al., 2004). In contrast, in structures with an open or atypical WPD loop conformation this water molecule was not observed or was significantly displaced, suggesting that it is a key part of the closure mechanism.

### A Secondary Substrate-Binding Pocket Is Present in Many PTPs

**Figure 4:**
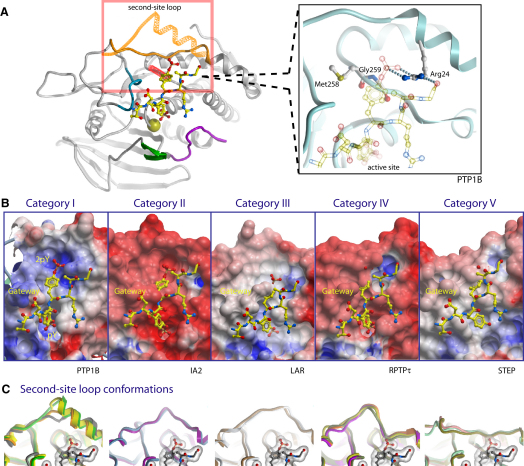
Secondary Substrate-Binding Pockets (A) Two extreme conformations of the second-site loop are shown (orange) from RPTPγ (extended helix) and HEPTP (closed in conformation). The catalytic cysteine is shown in a space-filling CPK representation, and loops are colored as follows: WPD (magenta), β5/β6 loop (green), and gateway (red). The dually pTyr phosphorylated insulin receptor peptide (from PDB: 1G1H) is shown superimposed (for reference only) to indicate the position of the secondary substrate-binding pocket. The positions of Arg24 and gateway residues Met258 and Gly259 of PTP1B are shown in an enlarged view. (B) Surface topology and electrostatic charge for the active site (pY), gateway region, and secondary pocket (2pY) are shown for each of the five categories with the dually pTyr phosphorylated insulin receptor peptide superimposed. (C) Representative second-site loop conformations are shown for each category (see also Supplemental Data). Category I: SHP2, BDP1, LYP; Category II: IA2, IA2β; Category III: LAR, RPTPσ; Category IV: PTPH1, MEG1, PTPD2, CD45; Category V: STEP, HEPTP, PCPTP1.

In order to determine whether other PTPs have a secondary substrate-binding pocket analogous to that found in PTP1B, we analyzed the structural topology and residue characteristics of this region. The presence of a secondary substrate-binding site cannot easily be predicted by sequence comparisons alone since it depends not only on the characteristics of the residues in this region but also on the conformation of the loop connecting helix α2′ and helix α1, which we term the “second-site loop.” The great diversity in conformations of this loop is shown (Figure 4). On the basis of these structural comparisons the PTPs can be grouped into five categories. In the first category are TCPTP, SHP1, SHP2, BDP1, LYP, PEST, PTPBAS, DEP1, MEG2, and GLEPP1, which have a “PTP1B-like” accessible pocket that harbors a basic residue corresponding to Arg24 of PTP1B (Figure 4 and Supplemental Data). This pocket has the potential to accommodate pY at the +1 position as in PTP1B or potentially other acidic or phosphorylated residues in positions N-terminal to the substrate pY. Also within this category are RPTPγ and RPTPβ, which have the secondary pocket with a basic residue albeit with bulky residues in the gateway region.

In the second category are PTPs in which both the gateway and the second-site loop are open and accessible as found in the R8 pseudophosphatase group (IA2, IA2β) but a cysteine residue occupies the position of Arg24. The third category is exemplified by the R2A group (LAR, RPTPσ, and RPTPδ) in which the gateway region contains bulky residues that block access to an open secondary pocket, with an aspartate residue in the position corresponding to Arg24. In the fourth category, both the gateway and secondary pocket are blocked and the inaccessible binding cavity harbors an aromatic residue or a proline in position of Arg24. The second-site loop assumes a twisted conformation in these phosphatases. This architecture is present in the subgroups NT5 (PTPH1, MEG1), NT6 (PTPD1, PTPD2), R1 (CD45), R2B (RPTPμ, RPTPκ, RPTPρ), and R4 (RPTPα, RPTPɛ). In the fifth category, the gateway is open and accessible while the secondary site is blocked by an aromatic or proline residue located in the closed secondary site loop. This scenario is present in the R7 group phosphatases (PCPTP, STEP, and HEPTP).

### Activity of PTPs toward Peptide Substrates

**Figure 5:**
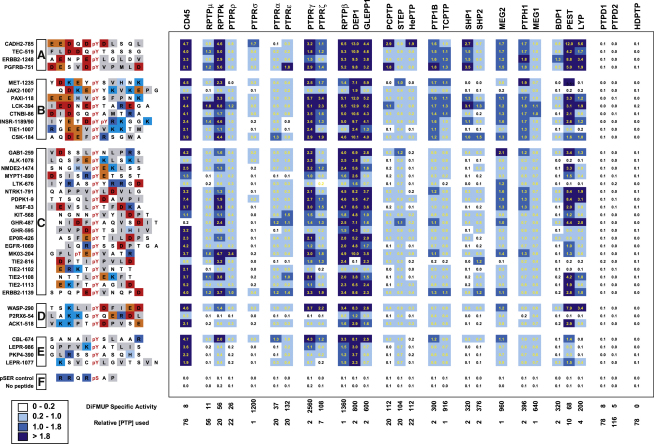
Analysis of PTP In Vitro Substrate Specificity Phosphatase activity of PTP catalytic domains against a panel of phosphopeptides derived from potential physiological substrates. Calculated reactions rates (Abs360/s) measured over control (a pSer-containing peptide and no peptide) have been color-coded with higher rates represented by darker shades of blue. Initial linear reaction rates were measured using the EnzCheck coupled continuous spectrophotometric assay over ∼5 min. Phosphopeptide names are derived from the SwissProt human gene name and the number of the pY residue. Sequences have been grouped based on sequence characteristics relative to the position of the pTyr: (A) N-terminal acidic; (B) N-terminal acidic and C-terminal basic; (C) mixed; (D) N-terminal basic and C-terminal acidic; (E) mixed basic; and (F) controls.

To assess enzymatic activity and substrate selectivity of PTP catalytic domains, we selected a panel of diverse phosphopeptides derived from known regulatory phosphorylation sites and assayed them against 28 highly purified PTPs (Figure 5). The peptides were grouped into acidic substrates, mixed acidic-basic substrates, and basic substrates based on the characteristics of residues in N- and C-terminal flanking regions of the phosphorylated site. Specific activity toward the general PTP substrate DiFMUP was measured and used to standardize the amount of protein for phosphopeptide assays. PTPs with particularly high enzymatic activity against generic substrates were RPTPσ and PTP subgroups R5, R3, NT1, NT2, NT3, NT4, and NT5. CD45 and members of subgroups R3 and R5 were extremely promiscuous and dephosphorylated most phosphopeptides with reasonable activity. The subgroups NT1, NT2, NT3, and NT5 exhibited a pronounced preference for acidic residues N-terminal to the phosphorylation site. In PTP1B this selectivity has been explained by substrate peptide interaction with Arg47 of the pY-recognition loop (Zhang, 2002).

In contrast, RPTPσ, the R7 group, and NT4 group PTPs were quite selective and showed a preference for a sequence derived from the phosphorylation site in N-cadherin (pY785), a reported substrate of RPTPσ (Siu et al., 2007). Surprisingly, the phosphatases PTPD1 and PTPD2 were inactive against the entire panel of phosphopeptides despite displaying a similar level of activity as CD45 toward DiFMUP. The phosphatase HDPTP was also inactive toward the generic substrate DiFMUP and the entire panel of phosphopeptides.

### Self-Association of PTPs In Vitro

**Figure 6:**
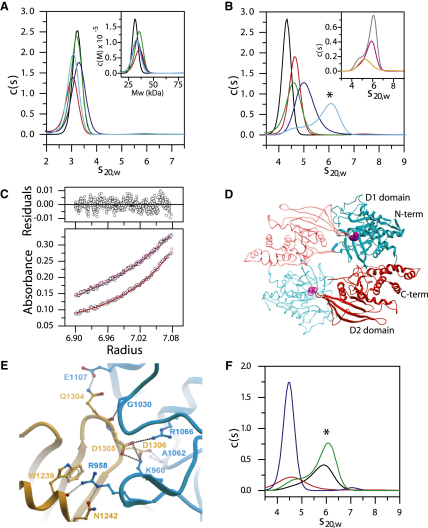
Self-Association of PTPs and Dimerization of RPTPγ (A) Sedimentation velocity AUC measurements of single-domain PTPs: IA2 (red); GLEPP1 (dark blue); DEP1 (green); IA2β (black); STEP (light blue). Differential sedimentation coefficient distribution, c(s), is plotted versus the apparent sedimentation coefficient corrected to water at 20°C, *s_20,w_*, together with the differential molecular weight distribution, c(M), versus molecular weight, M (inset). Experiments were conducted with a protein concentration of 0.8 mg/ml (∼24 μM). (B) Sedimentation velocity AUC measurements of tandem-domain RPTPs: RPTPα (black); CD45 (red); RPTPɛ (green); RPTPμ (dark blue); RPTPγ (light blue). Plotted data are as in (A). Inset shows experiments conducted with RPTPγ at protein concentrations of 0.2 (orange), 0.4 (magenta), and 0.8 (black) mg/ml. The dimer peak is indicated by an asterisk (^∗^). (C) Sedimentation equilibrium analysis of RPTPγ employing a rotor speed of 7500 (black) and 10,000 rpm (red). The solid line denotes a fitted curve resulting from global nonlinear regression analysis with a self-association model. The residuals for the fit are shown in the upper panel of the graph. The determined dissociation constant for the dimer was (K_D_) of 3.5 ± 0.3 μM. (D) Dimer interface in the crystal structure of RPTPγ. The two molecules interact in a head-to-toe orientation with the D1 domain (blue) of one molecule interacting with the D2 domain (red) of a second molecule. The catalytic cysteine (magenta) of the D1 domain is shown in a space-filling representation. (E) Details of the RPTPγ dimer interface. The backbone of the D1 domain from one molecule is colored blue and the backbone of the D2 domain from the interacting molecule is colored orange. H-bonds (black) and salt-bridges (gray) are depicted as dotted lines. See Supplemental Data for further details. (F) Disruption of the RPTPγ dimer interface by site-directed mutagenesis. The figure shows sedimentation velocity data using wild-type RPTPγ and RPTPγ dimer interface mutants. RPTPγ wild-type 0.8 mg/ml (green) and 0.4 mg/ml (black); RPTPγ-RKEE mutant 0.8 mg/ml (blue) and RPTPγ-DDKK mutant 0.4 mg/ml (red) are shown. The dimer peak is indicated by an asterisk (^∗^).

Dimerization of receptor PTPs has been proposed as a key mechanism of regulation that leads to inhibition of enzymatic activity. We have used analytical ultracentrifugation (AUC) to examine the catalytic domain oligomerization of single- and tandem-domain receptor PTPs from all of the major subgroups. Surprisingly, sedimentation velocity measurements showed that all single-domain RPTPs studied (IA2, IA2β, GLEPP1, DEP1, and STEP) were entirely monomeric in solution (Figure 6A), as were tandem-domain receptor PTPs (RPTPα, CD45, RPTPɛ, and RPTPμ) (Figure 6B). The determined molecular masses calculated from AUC velocity data ranged from 68.5 kDa to 74.2 kDa in agreement with the theoretical mass of monomeric tandem domains.

In contrast RPTPγ showed significant and concentration-dependent dimerization (Figure 6B). Sedimentation equilibrium confirmed that RPTPγ formed a stable dimer in solution. The experimental data fit well to a self-association model resulting in determination of a dissociation constant (K_D_) of 3.5 ± 0.3 μM. The correct masses for the monomeric and dimeric protein were obtained using this analysis and the residuals showed low systematic deviations (Figure 6C).

### RPTPγ Dimerizes in a Head-to-Toe Orientation

Analysis of the RPTPγ tandem-domain structure revealed that RPTPγ associated with a symmetry-related molecule as a “head-to-toe” dimer (i.e., with the D1 domain of one molecule interacting with the D2 domain of a second molecule and vice versa) (Figure 6D). The interaction between RPTPγ monomers involves extensive electrostatic interactions from the active site D1 domain of one RPTPγ molecule, visible as a highly electropositive pocket, interacting with an electronegative surface of the D2 from the other molecule. The dimer interface covers 1200 Å^2^ (∼5% of the total molecule surface area) and involves residues from the phosphotyrosine recognition loop, the P loop at the bottom of the active site cleft, and sheet β6, while the D2 domain interface involves the loop connecting strands β10–β11. Multiple hydrogen bonds and salt bridges are involved in the interaction between the two molecules (Supplemental Data). In this dimeric form, the active site of RPTPγ is occluded by the D2 domain of the interacting molecule, suggesting that dimerization in this conformation would prevent substrate access leading to suppression of enzymatic activity.

In order to determine whether the molecular mechanism of dimerization in solution correlates with that observed in the crystal structure, we mutated residues of the dimer interface from either the D1 or D2 domains (Figure 6E). In RPTPγ mutant “RKEE” residues, Arg958 and Lys960 of the D1 domain were mutated to glutamic acid, and in mutant “DDKK” residues, Asp1305 and Asp1306 of the D2 domain were mutated to lysine. Both the RKEE and DDKK mutants were entirely monomeric in solution, as assessed by velocity and equilibrium AUC analysis validating the head-to-toe dimerization model (Figure 6F).

## Discussion

This study provides structural information for more than 22 human PTP catalytic domains, thereby completing structural coverage for all major subgroups of the classical PTP family and enabling a comprehensive large-scale structural comparison. This resource provides a family-wide insight into catalytic (WPD) loop dynamics, self-association, and catalytic domain substrate specificity.

Structural comparisons identified an atypically open WPD loop conformation in LYP, GLEPP1, and STEP (Eswaran et al., 2006), suggesting a regulatory role in diverse family members. This conformation represents an inactive conformation in which the WPD aspartate is moved far out of the active site. Low crystallographic temperature factors observed in this loop region suggest that the atypically open conformation is stable, and multiple crystal forms ruled out that the WPD loop assumed this conformation as a result of crystal contacts. In protein kinases, inactive conformations and active site dynamics are crucial for the regulation of catalytic activity (Huse and Kuriyan, 2002). By analogy this conformation may represent a regulatory mechanism for PTPs and may find applications in the development of selective and potent inhibitors that target PTP inactive states.

### Secondary Substrate-Binding Pockets

The new structural data and analysis presented here suggest that a secondary substrate-binding pocket, similar to that found in PTP1B, is present also in SHP2, BDP1, LYP, SHP1, TCPTP, MEG2, PTPBAS, DEP1, and GLEPP1. Previous structural studies of PTPBAS also provided evidence of a secondary phosphotyrosine pocket (Villa et al., 2005). The basic pocket is also found in the phosphatases RPTPγ and RPTPβ; however, the topology of the region differs in these phosphatases in that the gateway region is inaccessible. The hypothesis that SHP1 and LYP have the potential to interact with phosphoproteins containing adjacent phosphotyrosines is consistent with reports that Zap-70 is a substrate of these PTPs (Pao et al., 2007). However, given that a relatively small number of proteins contain two adjacent phosphotyrosines, this pocket in other PTPs may interact with phosphothreonine, serine, or an acidic residue in a position N-terminal to the substrate pY. The secondary pocket of PTP1B, and more recently LYP, has been successfully exploited in the design of selective bidentate inhibitors (Yu et al., 2007). A similar inhibitor design strategy may also be used in the design of inhibitors of SHP2 for cancer (Chan et al., 2008) and may now be applied to a much wider subset of PTPs.

### Catalytic Activity

Our analysis of phosphatase activity using both a generic substrate and a panel of phosphopeptides represents a large set of comparable data across representative PTPs. The specific activity and phosphopeptide selectivity profile of each PTP correlate closely within their respective subgroups. It is therefore unlikely that the obtained data are influenced by the chosen domain boundaries or the presence of epitope tags in some proteins. In addition, all analyzed proteins had crystallization grade purity. The finding that specific activity of PTPs varies markedly across the PTP family implies that catalytic activity provides a mechanism of regulation in itself. All members of the R3 subgroup as well as RPTPγ were highly active, and it will be interesting to establish how this activity is regulated in vivo. In the case of RPTPγ, which had the highest enzymatic activity in our panel, the observed head-to-toe dimerization might provide such an inactivating mechanism. In contrast R1 group members (e.g., CD45) and R2B group had a more than two orders of magnitude lower enzymatic activity, suggesting that enhancement of activity by tight association with substrates in signaling complexes may be important for efficient signaling. RPTPα, RPTPɛ, and RPTPσ displayed a more limited peptide recognition capacity in vitro, which may be related to the presence of a bulky residue in the gateway region. RPTPσ showed a preference for a sequence derived from a phosphorylation site in N-cadherin (pY785), consistent with the recent identification of N-cadherin as an in vivo RPTPσ substrate (Siu et al., 2007).

Remarkably PTPD1, PTPD2, and HDPTP were completely inactive against all peptides, even at high enzyme concentrations. Mass spectrometry confirmed that the protein mass corresponds to the expected mass, ruling out oxidation as an explanation for the lack of activity. Also, analysis of multiple PTPD1, PTPD2, and HDPTP constructs over a pH range (pH 5.5–8.2) gave similar results and the crystal structure of PTPD2 (Barr et al., 2006) confirmed correct folding of the PTP domain. Examination of the protein sequence indicates that these phosphatases contain one or more atypical residues in the conserved phosphotyrosine recognition loop (KNRY), the WPD loop, and the phosphate-binding loop (Andersen et al., 2001). The lack of PTPD2 activity toward phosphopeptides is likely due to the absence of a tyrosine residue in the phosphotyrosine recognition loop that has the function of stabilizing binding of the substrate pY via a π-π stacking interaction. Both PTPD1 and HDPTP have a variation in the highly conserved WPD loop sequence where a glutamate replaces the conserved aspartate residue. It is likely that this sequence change is critical since this mutation has been shown to reduce enzymatic activity by three orders of magnitude in PTP1B (Flint et al., 1997), and the WPE loop sequence is found in several RPTP D2 domains with negligible catalytic activity. HDPTP has a further sequence change (alanine/serine) in the phosphate-binding loop that resembles the inactive pseudophosphatases IA2/IA2β.

Several earlier publications agree with our in vitro findings (Cao et al., 1998; Ogata et al., 1999; Jui et al., 2000), but studies demonstrating activity in vivo have also been reported. For instance phosphatase activity of PTPD1 has been shown to regulate EGFR and SRC signaling (Cardone et al., 2004) and β-catenin has been reported as a substrate of PTPD2 in endothelial cells (Wadham et al., 2003). These PTPs might therefore be activated by a mechanism that remains to be elucidated.

Previously it was reported that the phosphatase PTPRQ (PTPS31), which contains glutamate instead of aspartate in the WPD loop, dephosphorylates phospholipids in preference to phosphopeptides (Oganesian et al., 2003). Based on this report, we assayed PTPD1, PTPD2, and HDPTP against a number of phospholipid and inositol phosphates; however, none of the three phosphatases exhibited activity toward these potential substrates (data not shown). Thus, PTPD1, PTPD2, and HDPTP are highly specific for certain phosphotyrosine peptide substrates, act on so far unidentified substrates, or are intrinsically inactive phosphatases that function by noncatalytic means as has been described for IA2β (Mziaut et al., 2006).

### Regulation by Dimerization

Our structural analysis of single- and tandem-domain RPTPs together with biophysical studies and prior structural information from CD45 and LAR (Nam et al., 1999, 2005) reveal that PTP catalytic domains do not dimerize in solution under physiological buffer conditions and provide strong evidence against the long-held inhibitory wedge model. The inhibited dimeric state involving the N-terminal wedge, as defined in the RPTPα crystal structure, is not present in any other PTP structure. Moreover, analysis of tandem-domain structures showed that the orientation of D1 and D2 domains is highly conserved and is incompatible with the inhibitory wedge model due to a steric clash of D2 domains (Supplemental Data). In the present study we showed that this is also true for RPTPɛ, which is in the R4 subgroup together with RPTPα, the prototype of the inhibitory wedge model. All constructs used encompass the sequence corresponding to the N-terminal helix-turn-helix, i.e., the “N-terminal wedge,” of RPTPα D1. The secondary structure of this region (i.e., helix-turn-helix) is conserved in all classical PTPs, including RPTPγ, although amino acid residue conservation is low. The protein concentration (∼1 mg/ml, 15–30 μM) used in our studies corresponds to an estimate of the RPTP concentration in a membrane (∼10 μM). Although we conclude that PTP catalytic domains do not dimerize in solution, regulation of RPTPs by dimerization may occur in vivo through other means involving transmembrane domains, juxtamembrane regions (Gross et al., 2002), or extracellular ligands (Lee et al., 2007), proteolysis of the D1-D2 linker (Sonnenburg et al., 2003), or upon oxidation (Blanchetot et al., 2002; Groen et al., 2008; Tonks, 2005). In support of these data we observed dimerization of RPTPα under oxidizing conditions (Supplemental Data).

In contrast to other RPTPs, RPTPγ did self-associate strongly in solution and a dimeric head-to-toe arrangement of molecules was observed in the crystal structure. Mutation of residues from both the D1 and the D2 dimer interfaces abolished dimerization confirming that the molecular basis for the dimerization observed in solution correlates with the dimer model suggested by the crystal structure (Figure 6). Our model predicts that RPTPγ mutants would have greater catalytic activity than the wild-type at concentrations that promote dimerization; however direct comparison at high concentration was not feasible due to assay limitations. A head-to-toe dimerization model involving RPTPδ and RPTPσ has also been suggested based on yeast two-hybrid studies with individual D1 and D2 domains (Wallace et al., 1998). We did not analyze heterodimerization in this study; however, analysis of surface electrostatics suggests that the molecular interaction we observe for RPTPγ is not directly applicable to other RPTPs.

**Figure 7:**
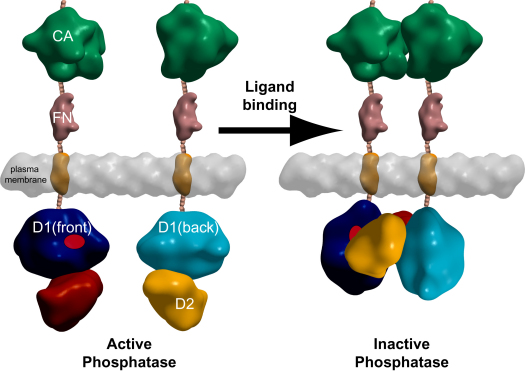
Schematic Model of RPTPγ Dimerization-Induced Inactivation The proposed transition of RPTPγ from monomer to dimer on ligand binding is shown. The carbonic anhydrase (CA), fibronection (FN), and intracellular tandem phosphatase (D1 and D2) domains are represented as low-resolution surfaces. Surface representations are based on PDB codes: 1JDO for the carbonic anhydrase domain, 2GEE for the fibronectin domain, and 2NLK for the tandem-phosphatase domain. In the monomeric state, the active site of RPTPγ (red) is accessible and the phosphatase is active. Ligand binding to the extracellular part of RPTPγ brings two molecules into close proximity and consequently the phosphatase domains dimerize in a head-to-toe arrangement as in the RPTPγ crystal structure with the D2 domain of one molecule blocking the active site (D1) from a second molecule, leading to suppression of phosphatase activity.

For RPTPζ, extracellular ligands have been identified including the heparin-binding growth factors, pleiotrophin, and the cytotoxin VacA secreted by *Helicobacter pylori* (Fujikawa et al., 2003; Maeda et al., 1999), and it has been reported that ligand binding induces dimerization and inactivation of RPTPζ resulting in an increase in tyrosine phosphorylation of its substrate proteins such as β-catenin, Fyn, and GIT1 (Fukada et al., 2006; Nakamura et al., 2003; Perez-Pinera et al., 2007). Given the high sequence identity between RPTPζ and RPTPγ it is likely that both molecules are regulated in a similar manner and key residues of the dimer interaction are conserved between these two phosphatases (Supplemental Data). In our AUC studies, the dual-domain of RPTPγ dimerizes with a dissociation constant (K_D_) of 3.5 ± 0.3 μM, which is within the estimated plasma membrane concentration of 10 μM based on an estimate of 10,000 RPTPγ molecules in a cell. It is likely that an equilibrium exists between the monomeric and dimeric states in the membrane, and ligand binding to extracellular regions would shift the equilibrium to the dimeric inactive state in which the active site is inaccessible. Our proposed regulatory model (Figure 7) does not involve reorganization of the D1-D2 domain interaction but requires flexibility in the linker between the transmembrane domain and the first D1 phosphatase domain. The 86 residue linker between the plasma membrane and start of the D1 domain is sufficiently long to allow for the required flex/turn to accommodate this model. However, future studies are necessary to establish this molecular mechanism in vivo as a form of inhibitory regulation.

## Experimental Procedures

### Protein Expression and Purification

Expression constructs were amplified and subcloned into pGEX-6P2 vector (Amersham Biosciences), which incorporates a PreScission protease cleavage site, for expression as glutathione-S-transferase fusions or into modified pET vectors (Supplemental Data). The modified pET vectors with a LIC cloning site incorporate an N-terminal 6× His tag (pLIC-SG1, pNIC28-Bsa4) with a TEV cleavage site (MHHHHHHSSGVDLGTENLYFQ^∗^SM) or a C-terminal 6× His tag (pNIC-CH) without a cleavage site (AHHHHHH). All constructs were verified by sequencing. Expression constructs were transformed into *E. coli* BL21(DE3) and proteins were purified according to previously described procedures (Eswaran et al., 2006; see also Supplemental Data). Mass spectrometry on an LC-ESI-MS-tof was used to confirm the identity of the purified protein. Information on individual proteins is compiled in the Supplemental Data.

### Enzymatic Assays

Phosphatase activity against phosphopeptides was measured using the EnzCheck (Invitrogen) continuous spectrophotometric assay (Webb, 1992). Reactions were measured in a 384 well plate in 80 μl containing 50 mM Tris-HCl, pH 7.4, 1 mM MgCl_2_, 50 mM NaCl, 1 mM DTT, 200 μM MESG (2-amino-6-mercapto-7-methylpurine riboside), 1 U/ml PNP, 125 μM of the phosphopeptide and PTP concentrations as shown in Figure 5. Absorbances were measured continuously at 360 nm using a Spectramax plate reader at room temperature and initial linear reaction rates were calculated over a 5 min reaction. Specific activity toward 6,8-difluoro-4-methylumbelliferyl phosphate (DiFMUP) was measured in 384 well plate format using a buffer containing 25 mM MOPS, pH 7, 50 mM NaCl, and 1 mM DTT and excitation and emission wavelengths of 355 nm and 460 nm, respectively (see Supplemental Data for further details).

### Analytical Ultracentrifugation

Sedimentation velocity experiments were carried out on a Beckman XL-I Analytical Ultracentrifuge. Protein samples were studied at concentrations of 0.2–0.8 mg/ml in 10 mM HEPES (pH 7.5), 150 mM NaCl, and 1 mM TCEP at 8°C, employing a rotor speed of 50,000 rpm. Absorbance data were analyzed with SEDFIT version 9.4 (Schuck, 2000) calculating c(s) distributions. Equilibrium experiments were performed at three protein concentrations (0.2, 0.4, and 0.8 mg/ml) and two centrifugation speeds (7500 rpm and 10,000 rpm), and data were evaluated by using the software package Ultraspin (Dimitry Veprintsev, MRC Centre for Protein Engineering).

### Crystallization and Structure Determination

Individual proteins were crystallized in sitting drops at either 4°C or 20°C. Crystals were cryoprotected and flash frozen, and X-ray diffraction data were collected at 100 K on beam lines X10SA at the Swiss Light Source (SLS), on beam line 14.1 at the Berliner Elektronenspeicherring-Gesellschaft für Synchrotronstrahlung (BESSY), and at a Rigaku FRE Superbright home source. Diffraction images were indexed and integrated using MOSFLM (Leslie, 1992) or DENZO in HKL2000 (Otwinowski and Minor, 1997) or XDS (Kabsch, 1993) and data were scaled using SCALA, SCALEPACK in HKL2000, or XSCALE, respectively. Structures were solved by molecular replacement using PHASER (McCoy et al., 2007) and were refined against maximum likelihood targets using restrained refinement and TLS protocols implemented in REFMAC (Murshudov et al., 1997). Iterative rounds of refinement were interspersed with manual rebuilding in COOT (Emsley and Cowtan, 2004). Additional information is compiled in the Supplemental Data and is also available at http://www.sgc.ox.ac.uk/research/phosphatases.

## Accession Numbers

The crystal structures reported in this paper have been deposited in the Protein Data Bank with the following PDB codes: 2OC3 (PTPN18, BDP1); 2JJD (PTPRE, RPTPɛ); 2PA5 (PTPN9, MEG2); 2B49 (PTPN3, PTPH1); 2I75 (PTPN4, MEG1); 3B7O (PTPN11, SHP2 D1); 2A8B (PTPRR, PCPTP1); 2QEP (PTPRN2, IA2β); 2P6X (PTPN22, LYP); 2BZL (PTPN14, PTPD2); 2BIJ (PTPN5, STEP); 2BV5 (PTPN5, STEP); 2CJZ (PTPN5, STEP-Cys/Ser mutant & pY); 2A3K (PTPN7, HePTP); 2C7S (PTPRK, RPTPκ); 2OOQ (PTPRT, RPTPρ); 2NLK (PTPRG, RPTPγ D1-D2); 2H4V (PTPRG, RPTPγ D1); 2AHS (PTPRB, RPTPβ); 2GJT (PTPRO, GLEPP1); 2NZ6 (PTPRJ, DEP1-Cys/Ser mutant); 2CFV (PTPRJ, DEP1-Trp/Ala mutant).

## Supplemental Data

Document S1. Supplemental Experimental Procedures, Five Figures, and Five Tables
