# Supplementary material for: Large-Scale Structural Analysis of the Classical Human Protein Tyrosine Phosphatome
Source: Cell. 2009 Jan 23;136(2):352–63. doi: 10.1016/j.cell.2008.11.038 (PMC2638020; doi:10.1016/j.cell.2008.11.038)
Supplement: Document S1. Supplemental Experimental Procedures, Five Figures, and Five Tables [file mmc1.pdf]

Cell, Volume *136*

Supplemental Data

Large-Scale Structural Analysis of the Classical Human Protein Tyrosine Phosphatome

Alastair J. Barr, Emilie Ugochukwu, Wen Hwa Lee, Oliver N. F. King, Panagis Filippakopoulos, Ivan Alfano, Pavel Savitsky, Nicola A. Burgess-Brown, Susanne Müller, Stefan Knapp

Supplemental Experimental Procedures

Protein Expression and Purification: cells were cultured in LB containing either ampicillin (100 μg/ml) or kanamycin (50 μg/ml) at 37 °C until the OD_{600} reached ~0.3 then the temperature was adjusted to 18 °C. Expression was induced for 12 h using 1 mM IPTG at an OD_{600} of 0.8. Cell pellets were resuspended in either 50mM HEPES pH 7.5, 500 mM NaCl, 5 mM imidazole, 5% glycerol, 0.5 mM TCEP for His tag fusion proteins or 50 mM Tris-HCl, pH 7.5, 250 mM NaCl, 10 mM DTT for GST fusion proteins, and cells were lysed using a high pressure cell disrupter. The lysate was cleared by centrifugation. For 6 x His fusions, supernatants were loaded onto a DEAE cellulose column, to remove nucleic acids, followed by a Ni^{2+}-NTA agarose column. Columns were washed with 50mM HEPES pH 7.5, 500 mM NaCl, 30 mM imidazole, 5% glycerol, 0.5 mM TCEP and eluted step-wise with a gradient of 50-250 mM imidazole. The 6 x His tag of certain constructs was cleaved by incubating the protein overnight with TEV protease at 4 °C. For GST fusions, the supernatants were loaded onto glutathione-sepharose, washed with 50 mM Tris-HCl, pH 7.5, 250 mM NaCl, 10 mM DTT and digested with PreScission protease overnight at 4 °C. Cleaved protein was eluted with three volumes of binding buffer. Proteins were further purified by gel-filtration

over Superdex-200 or by ion-exchange on HiTrap Q, and concentrated using Centricon concentrators (10 kDa cut-off).

Enzymatic Assays: Phosphatase activity against phospho-peptides was measured using the EnzCheck® (Invitrogen) continuous spectrophotometric assay which is based upon the purine nucleoside phosphorylase (PNP)-coupled assay reported by (Webb, 1992). Reactions were measured in a 384 well plate in 80 μl containing 50 mM Tris-HCl, pH 7.4, 1 mM $MgCl_2$, 50 mM NaCl, 1 mM DTT, 200 μM MESG (2-amino-6-mercapto-7-methylpurine riboside), 1 U/ml PNP, 125 μM of the phospho-peptide and PTP concentrations as shown in Figure 5. The concentration of each purified PTP enzyme used (ranging from 45 nM to 5 μM) was adjusted based on the specific activity of each enzyme towards DiFMUP. PNP and MESG concentrations were optimized to ensure that the phosphatase activity was rate limiting. Absorbances were measured continuously at 360 nm using a Spectramax plate reader at room temperature and initial linear reaction rates were calculated over a 5 minute reaction. Specific activity (fluorescence units/sec/fmole) towards 6,8-difluoro-4-methylumbelliferyl phosphate (DiFMUP) was measured in 384 well plate format using a buffer containing 25mM MOPS, pH 7, 50 mM NaCl and 1 mM DTT and an excitation and emission wavelengths of 355 nm and 460 nm, respectively.

Table S1

Crystallographic data and refinement statistics

**Data collection**	**2AHS (RPTPβ)**	**2GJT (GLEPP1)**	**2NZ6 (DEP1-C/S)**	**2CFV (DEP1-W/A)**
Space group	$P6_122$	$P6_5$	H3	H3
Cell dimensions [Å]	123.37, 123.37, 179.57	131.06, 131.06, 77.53	88.52, 88.52, 118.95	86.06, 86.06, 119.54
Cell angles [°]	90.00, 90.00, 120.00	90.00, 90.00, 120.00	90.00, 90.00, 120.00	90.00, 90.00, 120.00
Resolution [Å]	2.10	2.15	2.30	2.50
Total obs. (Unique, red.)	597804 (47341, 12.6)	231863 (41322, 5.6)	50144 (15274, 3.3)	43745 (11491, 3.76)
Completeness (outer shell)	99.2% (95.1%)	99.9% (99.4%)	99.2% (95.0%)	98.8% (91.2%)
R_{merge}	0.097	0.0786	0.089	0.06
I/σ (outer shell)	18.6 (2.50)	12.9 (2.87)	11.5 (2.70)	15.25 (4.18)
**Refinement**				
R_{work} (R_{free}) (%)	15.2 (20.6)	17.9 (23.4)	18.2 (22.8)	20.1 (25.0)
Protein atoms (water)	4502 (479)	4608 (213)	2393 (94)	2130 (25)
Rmsd bond length [Å]	0.013	0.017	0.011	0.012
Rmsd bond angle	1.337	1.587	1.291	1.296
Hetero groups:	CL, EDO, NA	CL	CL, NI, PO4	CL, NI
**Ramachandran**				
(allowed, disallowed) [%]	100.0, 0.0	99.6, 0.4	100.0, 0.0	99.6, 0.4

**Data collection**	**2C7S (RPTPκ)**	**2OOQ (RPTPρ)**	**2NLK (RPTPγ D1-D2)**	**2H4V (RPTPγ D1)**
Space group	$P4_32_12$	$P2_1$	$P2_12_12$	$P2_12_12_1$
Cell dimensions [Å]	91.37, 91.37, 108.42	37.19, 86.70, 91.12	118.51, 134.00, 59.08	74.85, 78.86, 121.76
Cell angles [°]	90.00, 90.00, 90.00	90.00, 99.89, 90.00	90.00, 90.00, 90.00	90.00, 90.00, 90.00
Resolution [Å]	1.95	1.80	2.40	1.55
Total obs. (Unique, red.)	228037 (32866, 6.1)	196449 (52802, 3.7)	156862 (37418, 4.2)	762141 (105096, 7.3)
Completeness (outer shell)	96.3% (87.7%)	100.0% (100.0%)	99.5% (98.6%)	100.0% (100.0%)
R_{merge}	0.0797	0.092	0.099	0.079
I/σ (outer shell)	11.14 (4.19)	16.0 (2.6)	14.2 (1.9)	16.0 (2.0)
**Refinement**				
R_{work} (R_{free}) (%)	20.0 (21.8)	14.5 (18.8)	23.7 (27.4)	16.1 (18.1)
Protein atoms (water)	2307 (138)	4464 (441)	4386 (150)	4645 (562)
Rmsd bond length [Å]	0.011	0.015	0.015	0.013
Rmsd bond angle	1.202	1.453	1.565	1.411
Hetero groups:	ACT	B3P, EDO, NA		ACT, CL, EDO, FLC, NA
**Ramachandran**				
(allowed, disallowed) [%]	100.0, 0.0	99.8, 0.2	100.0, 0.0	99.7, 0.3

Data collection	2BIJ (STEP)	2BV5 (STEP)	2CJZ (STEP-C/S & pY)	2A3K (HePTP)
Space group	$P2_12_12_1$	$P2_12_12_1$	$P2_12_12_1$	$P2_12_12_1$
Cell dimensions [Å]	51.81, 64.32, 101.08	39.96, 64.01, 136.15	52.34, 64.35, 100.78	39.13, 80.97, 100.39
Cell angles [°]	90.00, 90.00, 90.00	90.00, 90.00, 90.00	90.00, 90.00, 90.00	90.00, 90.00, 90.00
Resolution [Å]	2.05	1.80	1.7	2.55
Total obs. (Unique, red.)	107431 (20825, 4.71)	167346 (32877, 4.97)	130114 (37692, 3.4)	38503 (10922, 3.5)
Completeness (outer shell)	95.0% (97.4%)	98.7% (95.4%)	98.5% (91.6%)	99.4% (96.6%)
R_{merge}	0.0867	0.0823	0.0713	0.128
I/σ (outer shell)	11.41 (3.05)	12.90 (2.54)	11.92 (3.07)	9.22 (2.05)
**Refinement**				
R_{work} (R_{free}) (%)	21.2 (26.4)	16.4 (20.1)	17.5 (20.5)	21.9 (27.5)
Protein atoms (water)	2272 (103)	2296 (271)	2204 (213)	2134 (32)
Rmsd bond length [Å]	0.015	0.011	0.008	0.011
Rmsd bond angle	1.484	1.322	1.107	1.270
Hetero groups:	SO4	GOL, SO4	EDO, PTR	PO4
**Ramachandran**				
(allowed, disallowed) [%]	99.6, 0.4	99.7, 0.3	99.6, 0.4	99.2, 0.8

Data collection	2A8B (PCPTP1)	2QEP (IA2β)	2P6X (LYP)	2BZL (PTPD2)
Space group	$P2_12_12$	$P2_12_12$	$P2_1$	$P3_221$
Cell dimensions [Å]	63.19, 74.10, 62.33	131.54, 136.55, 35.75	60.96, 48.44, 119.81	87.64, 87.64, 77.11
Cell angles [°]	90.00, 90.00, 90.00	90.00, 90.00, 90.00	90.00, 102.84, 90.00	90.00, 90.00, 120.00
Resolution [Å]	2.30	2.50	1.90	1.65
Total obs. (Unique, red.)	46787 (13031, 3.6)	79716 (23096, 3.5)	201242 (54178, 3.7)	288670 (41374, 6.73)
Completeness (outer shell)	97.0% (98.4%)	99.5% (99.7%)	100.0% (100.0%)	99.5% (99.1%)
R_{merge}	0.105	0.139	0.080	0.0939
I/σ (outer shell)	10.5 (3.5)	9.1 (1.9)	13.1 (3.3)	15.08 (2.05)
**Refinement**				
R_{work} (R_{free}) (%)	19.2 (25.6)	23.6 (28.6)	16.4 (21.7)	18.5 (21.7)
Protein atoms (water)	2220 (132)	4413 (39)	4853 (373)	2268 (244)
Rmsd bond length [Å]	0.011	0.011	0.018	0.008
Rmsd bond angle	1.317	1.310	1.518	1.167
Hetero groups:	CL	CL	CL, EDO	EDO, SO4
**Ramachandran**				
(allowed, disallowed) [%]	99.6, 0.4	99.6, 0.4	100.0, 0.0	99.6, 0.4

Data collection	2PA5 (MEG2)	2B49 (PTPH1)	2I75 (MEG1)	3B7O (SHP2 D1)
Space group	P1	C2	$P4_12_12$	$C222_1$
Cell dimensions [Å]	39.97, 57.43, 66.45	62.67, 61.24, 75.89	66.07, 66.07, 144.51	44.813, 86.496, 166.555
Cell angles [°]	77.44, 78.22, 80.41	90.00, 101.21, 90.00	90.00, 90.00, 90.00	90.00, 90.00, 90.00
Resolution [Å]	1.60	1.54	2.45	1.60
Total obs. (Unique, red.)	142225 (71616, 2.0)	190501 (41752, 4.6)	74276 (11901, 6.2)	174991 (43184, 4.1)
Completeness (outer shell)	96.9% (95.3%)	99.7% (99.1%)	95.6% (98.6%)	99.9% (100%)
R_{merge}	0.063	0.085	0.167	0.092
I/σ (outer shell)	9.0 (2.1)	13.7 (2.0)	10.2 (1.8)	13.3 (2.0)
**Refinement**				
R_{work} (R_{free}) (%)	15.6 (18.8)	19.9 (23.0)	22.4 (29.1)	17.009 (20.897)
Protein atoms (water)	4675 (410)	2151 (229)	2089 (51)	2215 (241)
Rmsd bond length [Å]	0.014	0.011	0.012	0.017
Rmsd bond angle	1.434	1.402	1.347	1.593
Hetero groups:	CL, EDO, SCN		SO4	MLT
**Ramachandran**				
(allowed, disallowed) [%]	99.7, 0.3	99.6, 0.4	99.6, 0.4	99.6, 0.4

Data collection	2OC3 (BDP1)	2JJD (RPTPε)
Space group	$P2_1$	$P2_1$
Cell dimensions [Å]	46.34, 63.76, 48.99	126.02, 123.62, 219.12
Cell angles [°]	90.00, 102.64, 90.00	90.00, 91.13, 90.00
Resolution [Å]	1.50	3.20
Total obs. (Unique, red.)	162935 (44277, 3.7)	373908 (109786, 3.4)
Completeness (outer shell)	99.3% (95.2%)	98.7% (92.4%)
R_{merge}	0.065	0.145
I/σ (outer shell)	12.4 (3.4)	9.0 (1.5)
**Refinement**		
R_{work} (R_{free}) (%)	15.4 (18.4)	22.1 (25.6)
Protein atoms (water)	2228 (277)	23738 (12)
Rmsd bond length [Å]	0.014	0.010
Rmsd bond angle	1.471	1.169
Hetero groups:		CL
**Ramachandran**		
(allowed, disallowed) [%]	99.6, 0.4	99.2, 0.8

Figure S1

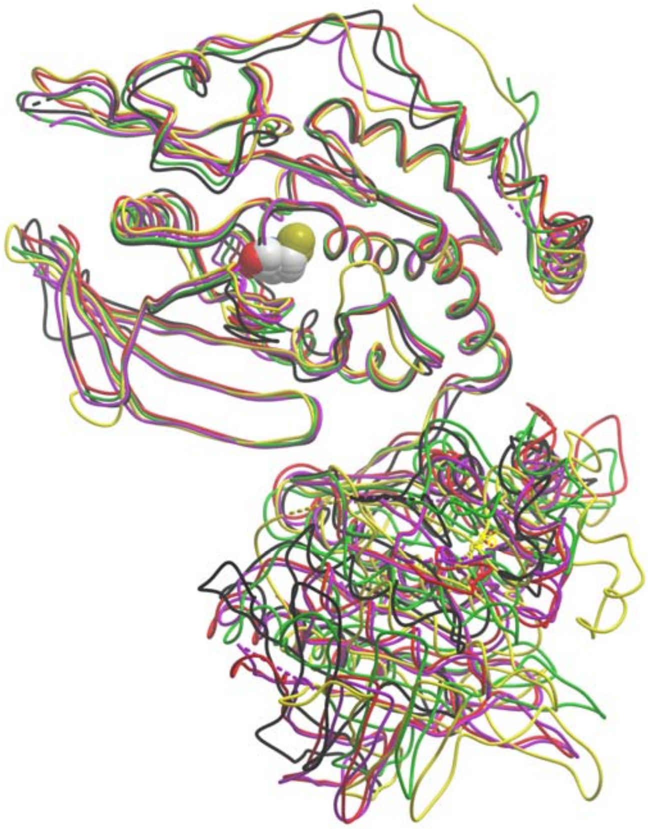

Figure S1: Superimposition of tandem domain RPTP structures. Superimposition of available tandem domain receptor PTP structures reveals that the orientation of D1 and D2 domains is highly conserved. RPTPγ (Black, PDB: 2NLK); CD45 (Yellow, PDB: 1YGR); LAR (Red, PDB: 1LAR); RPTPσ (Green, PDB: 2FH7) and RPTPε (Magenta, PDB: 2JJD). The catalytic cysteine of D1 domain is shown as CPK representation for orientation.

Table S2

Residues involved in the D1-D2 interface of tandem domain RPTP structures

RPTPγ (PTPRG)	D1 Domain	D2 Domain
**Number of Residues**		
interface	19 (6.5%)	23 (8.8%)
surface	263 (90.4%)	235 (89.7%)
total	291 (100%)	262 (100%)
**Solvent-accessible area, Å^2**		
interface	779.6 (5.5%)	722.9 (6.0%)
total	14277.1 (100%)	12063.6 (100.0%)
**Hydrogen Bonds**		
	Thr1040 [OG1]	Gln1381 [NE2]
	Ala987 [N]	Tyr1214 [O]
	Arg1044 [NH2]	Glu1126 [OE1]
	Arg1044 [NH2]	Glu1374 [OE1]
**Salt Bridges**		
	Arg1044 [NH2]	Glu1126 [OE1]
	Arg1044 [NH2]	Glu1374 [OE1]

CD45 (PTPRC)	D1 Domain	D2 Domain
**Number of Residues**		
interface	19 (6.5%)	19 (6.7%)
surface	268 (91.8%)	260 (92.2%)
total	292 (100%)	282 (100%)
**Solvent-accessible area, Å^2**		
interface	694.0 (5.1%)	709.2 (5.1%)
total	13645.1 (100%)	13784.2 (100.0%)
**Hydrogen Bonds**		
	Asp766 [OD1]	Lys1003 [NZ]
	Arg811 [NE]	Glu894 [OE1]
	Arg811 [NH2]	Glu894 [OE2]
	Tyr767 [OH]	Glu1167 [OE2]
**Salt Bridges**		
	Asp766 [OD1]	Lys1003 [NZ]
	Arg811 [NE]	Glu894 [OE1]
	Arg811 [NH2]	Glu894 [OE2]

RPTPσ (PTPRS)	D1 Domain	D2 Domain
**Number of Residues**		
interface	18 (6.3%)	22 (7.8%)
surface	248 (87.0%)	253 (89.4%)
total	285 (100%)	283 (100%)
**Solvent-accessible area, Å^2**		
interface	742.5 (5.6%)	666.3 (4.9%)
total	13285.0 (100%)	13460.6 (100.0%)
**Hydrogen Bonds**		
	Glu1562 [OE1]	Ser1719 [OG]
	Ala1527 [N]	Tyr1744 [O]

LAR (PTPRF)	D1 Domain	D2 Domain
**Number of Residues**		
interface	20 (7.2%)	22 (7.6%)
surface	247 (88.8%)	259 (89.9%)
total	278 (100%)	288 (100%)
**Solvent-accessible area, Å^2**		
interface	661.8 (5.1%)	621.2 (4.6%)
total	12935.4 (100%)	13574.4 (100.0%)
**Hydrogen Bonds**		
	Glu1495 [OE1]	Arg1629 [NH2]
	Tyr1496 [OH]	Arg1629 [NH2]
	Ala1581 [O]	Gly1585 [N]
	Ala1460 [N]	Tyr1677 [O]
**Salt Bridges**		
	Glu1495 [OE1]	Arg1629 [NH2]
	Arg1506 [NH1]	Glu1836 [OE2]

Figure S2

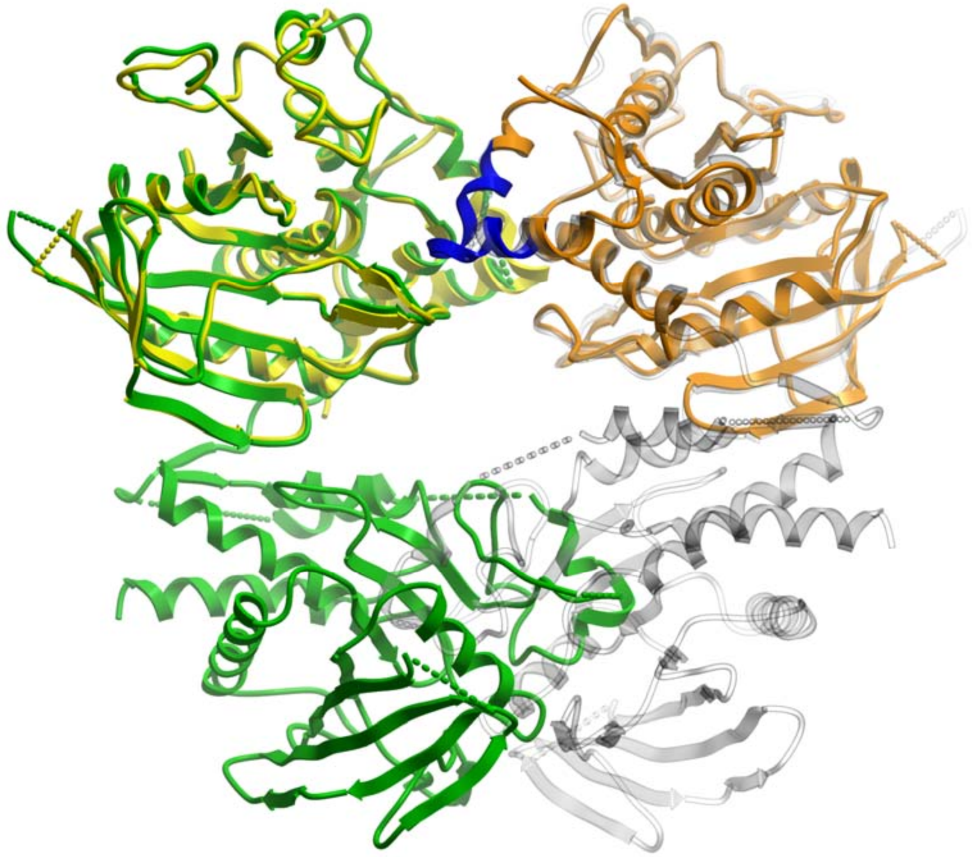

Figure S2: Theoretical arrangement of tandem domain RPTPs according to the ‘inhibitory wedge’ model. Two RPTPα D1 domains (yellow and orange) are shown in the dimeric form found in the crystal structure (PDB: 1YFO). The N-terminal inhibitory wedge is shown in blue blocking the active site of the opposing monomer. The structure of the tandem domain RPTPε (PDB: 2JJD) has been superimposed on each RPTPα D1 domain (green and grey shadow) revealing a steric clash of D2 domains making this conformation involving the inhibitory wedge impossible.

Table S3

Details of the RPTPγ head to toe dimer interface (PDB: 2NLK)

	Molecule A
**Number of Atoms**	
Interface (%)	132 (3.0%)
Surface (%)	2323 (53.0%)
Total (%)	4386 (100.0%)
**Number of Residues**	
Interface (%)	38 (6.9%)
Surface (%)	498 (90.1%)
Total (%)	553 (100.0%)
**Solvent-accessible area, Å^2**	
Interface / monomer (%)	1217.8 (4.9%)
total (%)	24838.2 (100.0%)

**Hydrogen bonds**			
	**Molecule A**	**Distance [Å]**	**Molecule B**
1	A:ARG 958[NE]	2.82	A:ASN1242[OD1]
2	A:ARG 958[NH2]	2.79	A:TRP1239[O]
3	A:GLY1030[N]	2.96	A:GLN1304[O]
4	A:ALA1062[N]	2.84	A:ASP1306[OD2]
5	A:GLU1107[OE1]	2.68	A:GLN1304[NE2]
**Salt bridges**			
1	A:LYS 960[NZ]	3.05	A:ASP1305[OD1]
2	A:LYS 960[NZ]	2.88	A:ASP1305[OD2]
3	A:LYS 960[NZ]	2.62	A:ASP1306[OD1]
4	A:ARG1066[NH2]	3.48	A:ASP1305[OD1]

Figure S3

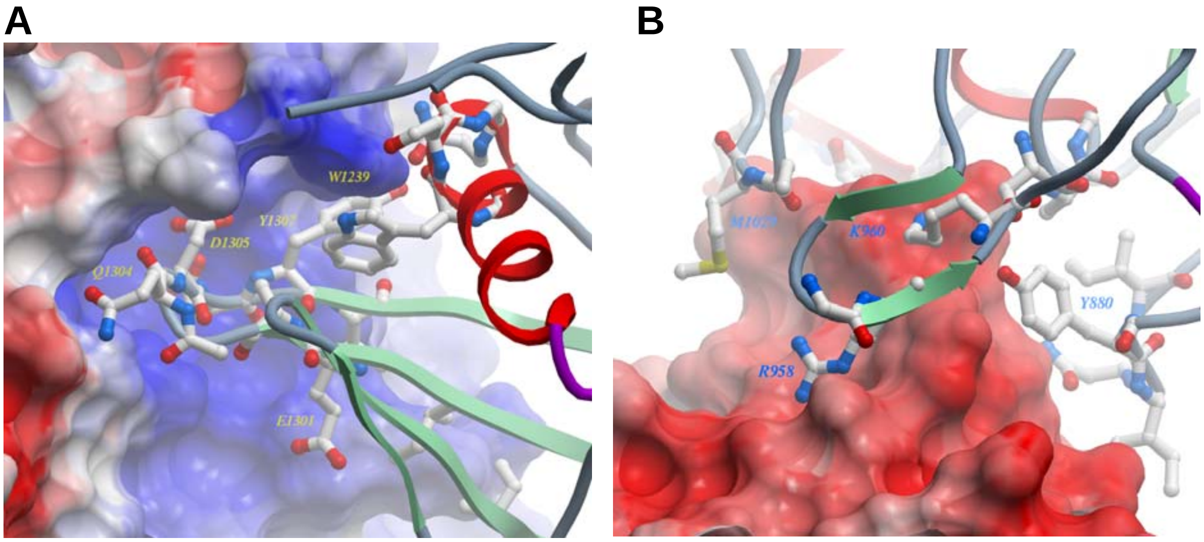

Figure S3: Details of the RPTPγ dimer interface. (A) The surface of the RPTPγ D1 domain and residues from the D2 domain (loop β10-β11) of an interacting molecule in the dimeric form are shown. (B) The surface of the RPTPγ D2 domain and residues from the D1 domain (sheet β6) of an interacting molecule in the dimeric form are shown

Figure S4

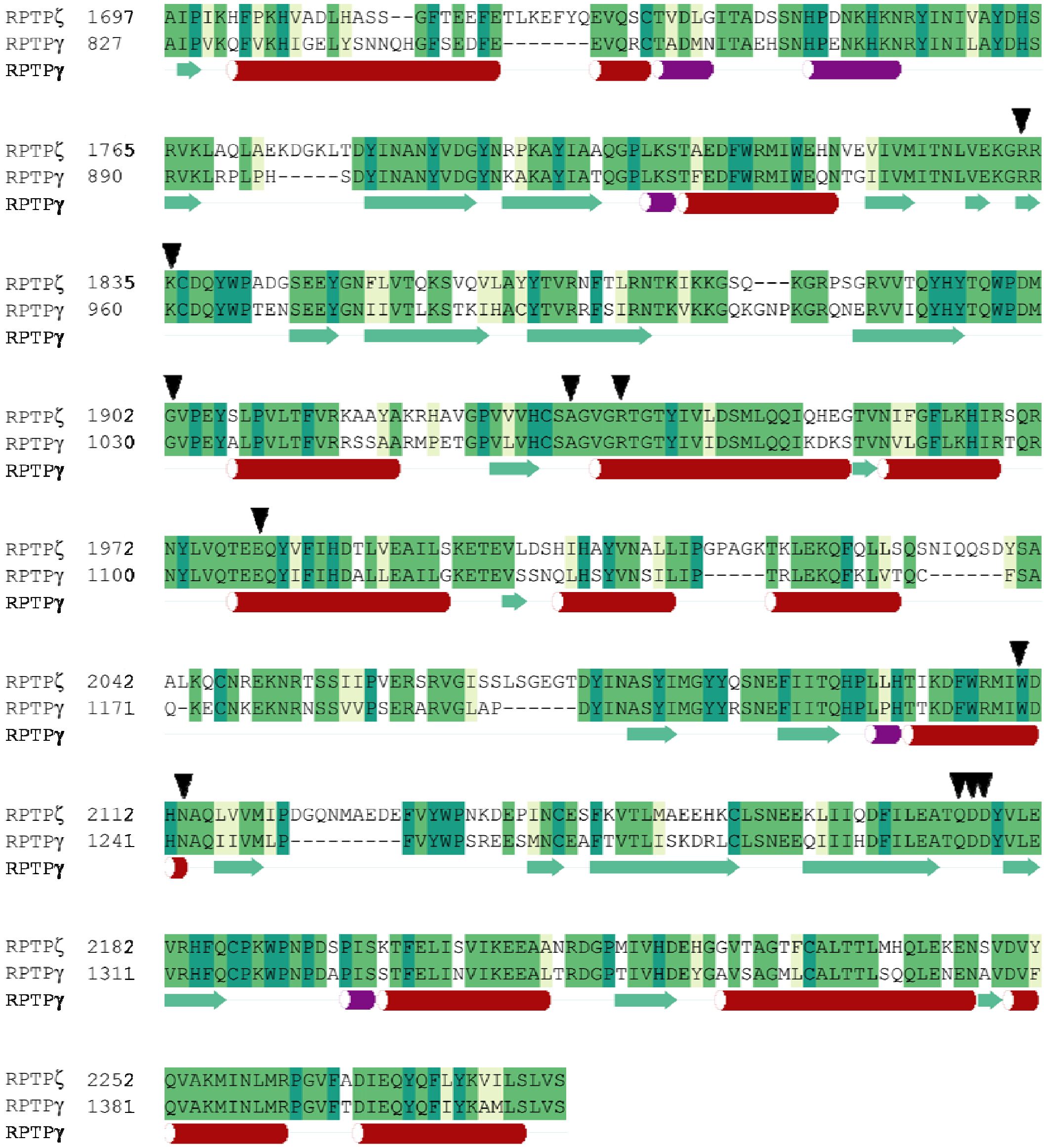

Figure S4: Sequence aligment of RPTPγ and RPTPζ showing conservation of residues involved the dimer interface (indicated by arrows).

Figure S5

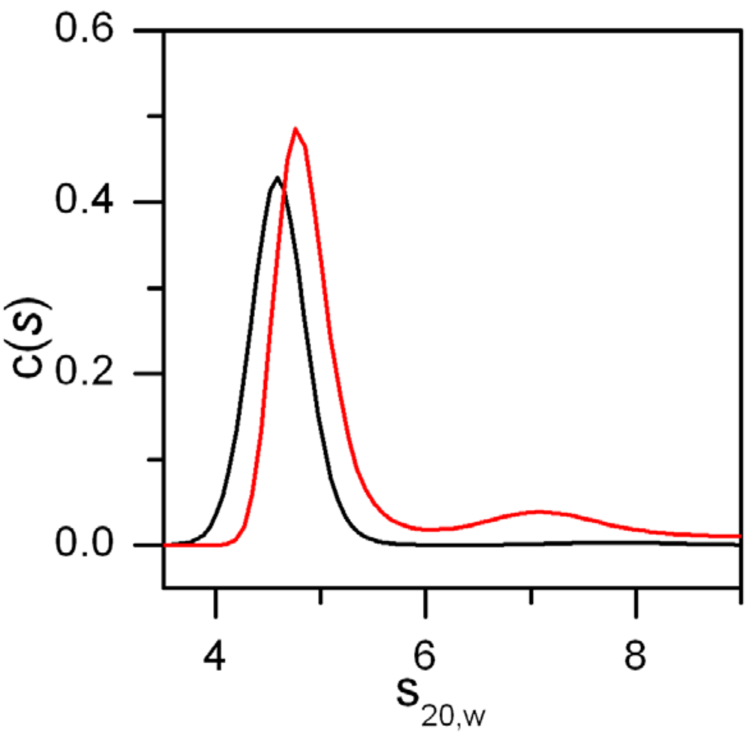

Figure S5: Effect of oxidation on the oligomeric state of RPTPα.

Sedimentation velocity AUC measurements using RPTPα (black) and RPTPα treated with H_20_2 (5 μM) for 30 minutes (red). Experiments were conducted with a protein concentration of 0.5 mg/ml for control RPTPα and 0.8 mg/ml for H_20_2 treated RPTPα. The data show that, in agreement with other reports, oxidation induces dimerization as assessed by AUC; however, higher concentrations of H_20_2 (25 μM) led to significant formation of oligomers up to a large molecular weight indicative of protein aggregation (data not shown).

Table S4

Templates used for homology models of PTPs

PTP homology models			
**PTP Model**	**Swiss Prot ID**	**PTP Template**	**Template PDB code**
PTPN12	Q05209	PTPN22	2P6X
PTPN20	Q4JDL3	PTPN13	1WCH
PTPN21	Q16825	PTPN14	2BZL
PTPRU	Q92729	PTPRS	2FH7
PTPRM (D2)	P28827	PTPRS (D2)	2FH7
PTPRK (D2)	Q15262	PTPRS (D2)	2FH7
PTPRT (D2)	O14522	PTPRF (D2)	1LAR
PTPRH	Q9HD43	PTPRB	2AHS
PTPRQ	Q9UMZ3	PTPRO	2GJT
PTPRZ	P23471	PTPRG	2NLK
PTPN23	Q9H3S7	PTPN2	1L8K

Table S5

Secondary Site loop conformations and gateway residues

PTP	Gateway Residues	Gateway Access	Secondary site loop Access	Secondary site loop [Arg24]	Secondary site loop Conformation	Category
SHP2	S G	0	open	Q	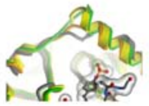	I
BDP1	P A	1	open	Q		I
LYP	P S	1	open	K		I
RPTPγ	N Y	3	open	Q		
SHP1	S G	0	open	Q		I
PEST	H S	n/a	n/a	R		
RPTPζ	N Y	3	open	Q		
PTP1B	M G	1	open	R	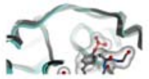	I
TCPTP	M G	1	open	R		I
MEG2	A F	1	open	R		I
BAS	H G	1	open	Q		I
TYP	S G	n/a	n/a	Q		
HDPTP	K H	n/a	n/a	Q		
RPTPβ	V H	3	open	K	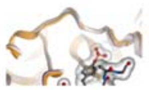	
DEP1	P L	2	open	K		I
GLEPP1	M S	2	open	K		I
SAP1	V L	n/a	n/a	S		
PTPS31	M C	n/a	n/a	P		
IA2	P G	0	open	C	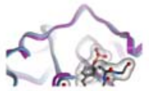	II
IA2β	P G	0	open	C		II
RPTPδ	N Y	3	open	D	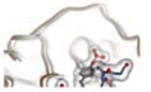	III
LAR	N Y	3	open	D		III
RPTPσ	N Y	3	open	D		III
RPTPκ	I N	2	closed	F	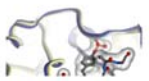	IV
RPTPμ	V N	2	closed	F		IV
RPTPτ	V N	2	closed	P		IV
RPTPλ	V N	n/a	n/a	F		
PTPH1	A M	3	closed	Y	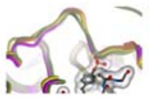	IV
MEG1	A M	3	closed	Y		IV
PTPD2	M F	3	closed	P		IV
CD45	C L	2	closed	P		IV
PTPD1	M M	n/a	n/a	L		
PCPT1	G G	0	closed	P	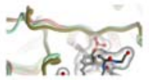	V
STEP	G G	0	closed	P		V
HEPTP	G G	0	closed	P		V
RPTPα	C Q	3	closed	P	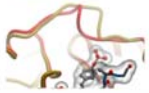	IV
RPTPε	P Q	3	closed	P		IV

Table S5: PTPs have been grouped into five categories according to accessibility via the gateway region, the second site loop conformation and characteristics of the residue corresponding to Arg24 of PTP1B. Accessibility via the gateway based on analysis of the structures is indicated via a score from 0 to 3 (0 = accessible gateway; 3 = hindered

gateway) and the conformation of the second site loop is indicated. Analysis was carried out on PTP crystal structures (black) and homology models (red).
